# Comparison of MALDI-TOF-MS and RP-HPLC as Rapid Screening Methods for Wheat Lines With Altered Gliadin Compositions

**DOI:** 10.3389/fpls.2020.600489

**Published:** 2020-12-04

**Authors:** You-Ran Jang, Kyoungwon Cho, Sewon Kim, Jae-Ryeong Sim, Su-Bin Lee, Beom-Gi Kim, Yong Q. Gu, Susan B. Altenbach, Sun-Hyung Lim, Tae-Won Goo, Jong-Yeol Lee

**Affiliations:** ^1^National Institute of Agricultural Sciences, RDA, Jeonju, South Korea; ^2^Department of Biotechnology, College of Agriculture and Life Sciences, Chonnam National University, Gwangju, South Korea; ^3^USDA-ARS, Western Regional Research Center, Albany, CA, United States; ^4^Division of Horticultural Biotechnology, Hankyong National University, Anseong, South Korea; ^5^Department of Biochemistry, School of Medicine, Dongguk University, Gyeongju, South Korea

**Keywords:** gliadin profiling, chromosomal assignment, aneuploid lines, MALDI-TOF-MS, RP-HPLC, immunogenic potential

## Abstract

The wheat gliadins are a complex group of flour proteins that can trigger celiac disease and serious food allergies. As a result, mutation breeding and biotechnology approaches are being used to develop new wheat lines with reduced immunogenic potential. Key to these efforts is the development of rapid, high-throughput methods that can be used as a first step in selecting lines with altered gliadin contents. In this paper, we optimized matrix-assisted laser desorption/ionization time-of-flight mass spectrometry (MALDI-TOF-MS) and reversed-phase high-performance liquid chromatography (RP-HPLC) methods for the separation of gliadins from *Triticum aestivum* cv. Chinese Spring (CS). We evaluated the quality of the resulting profiles using the complete set of gliadin gene sequences recently obtained from this cultivar as well as a set of aneuploid lines in CS. The gliadins were resolved into 13 peaks by MALDI-TOF-MS. α- or γ-gliadins that contain abundant celiac disease epitopes and are likely targets for efforts to reduce the immunogenicity of flour were found in several peaks. However, other peaks contained multiple α- and γ-gliadins, including one peak with as many as 12 different gliadins. In comparison, separation of proteins by RP-HPLC yielded 28 gliadin peaks, including 13 peaks containing α-gliadins and eight peaks containing γ-gliadins. While the separation of α- and γ-gliadins gliadins achieved by RP-HPLC was better than that achieved by MALDI-TOF-MS, it was not possible to link peaks with individual protein sequences. Both MALDI-TOF-MS and RP-HPLC provided adequate separation of ω-gliadins. While MALDI-TOF-MS is faster and could prove useful in studies that target specific gliadins, RP-HPLC is an effective method that can be applied more broadly to detect changes in gliadin composition.

## Introduction

Wheat (*Triticum aestivum* L.) is a major staple cereal grain consumed by humans worldwide and a major source of protein in the diet. The gluten proteins comprise about 70% of the total grain protein (Shewry and Halford, 2002). Gluten proteins consist of 70–100 different proteins that play a major role in the dough mixing properties of flours and determine their suitability for bread-making (Shewry et al., 2003). Some gluten proteins also trigger severe diseases in humans, including celiac disease (CD), and food allergies (Biagi et al., 1999; Matsuo et al., 2004, 2005; Battais et al., 2005a; Bittner et al., 2008; Sollid et al., 2012). Gluten proteins are traditionally classified into glutenins and gliadins (Shewry and Tatham, 1990). The polymeric glutenins consist of high-molecular-weight glutenin subunits (HMW-GS) and low-molecular-weight glutenin subunits (LMW-GS) that are linked by disulfide bonds. Gliadins are monomeric proteins that account for approximately 40% of the gluten proteins and are particularly immunogenic (Battais et al., 2005a). Gliadins are generally separated into four complex classes, α-, γ-, δ-, and ω-gliadin, based on their electrophoretic mobility in acid polyacrylamide gel electrophoresis (A-PAGE) (Anderson et al., 2012). The ω-gliadins are further divided into ω-5 gliadins and ω-1,2 gliadins on the basis of both size and repetitive motifs. A number of epitopes that trigger the food allergy wheat-dependent exercise-induced anaphylaxis (WDEIA) have been identified in ω-5 gliadins (Battais et al., 2005b), while α-, γ-, and ω-1,2 gliadins contain clusters of epitopes that are active in celiac disease (Tye-Din et al., 2010; Sollid et al., 2012).

γ-, δ-, and ω-gliadins are encoded at the *Gli-1* loci on the short arms of the group 1 homeologous chromosomes in hexaploid wheat and linked to the LMW-GS, while the α-gliadins are encoded at the *Gli-2* loci on the short arms of the group six chromosomes. Recent genome sequencing efforts in the reference wheat Chinese Spring (CS) revealed the complexity of the gluten protein families and a complete set of gliadin and LMW-GS genes including 47 α-gliadin, 14 γ-gliadin, five δ-gliadin, 19 ω-gliadin, and 17 LMW-GS genes was assembled and annotated [Huo et al., 2018a,b; International Wheat Genome Sequencing Consortium (IWGSC) et al., 2018]. Of these, genes for 26 α-gliadins, 11 γ-gliadins, two δ-gliadins, seven ω-gliadins, and 10 LMW-GS encode full-length proteins. Transcriptomic studies further revealed wide ranges of expression levels for individual genes within the families. Additionally, Altenbach et al. (2020) used two-dimensional gel electrophoresis (2-DE) combined with tandem mass spectrometry (MS/MS) to link individual protein spots in a total protein extract from CS flour to 16 of 26 α-gliadin, 10 of 11 γ-gliadin, one of two δ-gliadin, and six of seven ω-gliadin genes. Most of the genes that were not associated with protein spots encoded proteins that were very similar to other proteins or were expressed at low levels in transcriptomic experiments.

In efforts to reduce the immunogenic potential of wheat flour, many research groups are now using classical breeding methods or biotechnology techniques to eliminate gliadins that contain immunogenic sequences from wheat flour. Mutation breeding using ethyl methanesulfonate (EMS) or γ ray-irradiation, gene silencing by RNA interference (RNAi), and genome editing techniques such as CRISPR/Cas9 can be used to inactivate or delete single genes (EMS, CRISPR, and RNAi), multiple homologous genes (CRISPR, RNAi) or blocks of genes at particular chromosomal locations (γ ray-irradiation). Critical for these efforts is the availability of high-throughput screening methods that can be used as a first step in selecting lines with altered gliadin contents.

In this study, we optimized matrix-assisted laser desorption/ionization time-of-flight mass spectrometry (MALDI-TOF-MS) and reversed-phase high-performance liquid chromatography (RP-HPLC) methods for the analysis of gliadins. By using the reference wheat CS for this study, we were able to take into account the recently published set of gliadin sequences to determine whether the methods have sufficient resolution to reveal the complexity of this group of proteins. In addition, the analysis of aneuploid lines from CS made it possible not only to confirm the chromosomal locations of gliadins found in different peaks of the chromatograms, but also to determine whether lines that are missing regions of chromosomes with multiple gliadin genes could be distinguished. In this paper, we discuss the advantages and limitations of MALDI-TOF-MS and RP-HPLC for use in early screening experiments.

## Materials and Methods

### Plant Materials

Chinese Spring (*Triticum aestivum* L.) and its group 1 and 6 aneuploid lines were kindly provided by National Bioresource Project, Japan. The plants were grown and harvested at National Institute of Agricultural Sciences, Jeonju, South Korea in 2017.

### Extraction of Gliadin and Glutenin

For MALDI-TOF-MS analysis, gliadin was prepared as described by Dziuba et al. (2014). Thirty mg of flour was mixed with 150 μl 0.15 M NaCl solution and shaken for 2 h. After centrifugation at 12,000 rpm for 10 min, the supernatant containing albumin/globulin was discarded. The gliadin in the pellet was dissolved and extracted with 150 μl of 60% EtOH for 2 h. After centrifugation at 12,000 rpm for 5 min, 100 μl of the supernatant was moved to a new tube and stored at 4°C.

For RP-HPLC analysis, gliadins were prepared as described by DuPont et al. (2000). One-hundred mg of flour was dissolved in 1 ml of 70% EtOH and shaken for 2 h at room temperature. Gliadin proteins were extracted with 70% EtOH or 70% EtOH containing 0.15% NaCl. After centrifugation at 12,000 rpm for 10 min, 1 ml of the supernatant fraction was transferred to a new 1.5-ml tube and freeze-dried for 3 h. The dried gliadins were stored at −80°C until use.

For detection of contaminated LMW-GS peaks in RP-HPLC analysis of the gliadin fraction, glutenins were prepared as described by Singh et al. (1991). One-hundred mg of flour was extracted with 5 ml of 50% propanol with incubation for 30 min at 65°C, followed by centrifugation for 5 min at 10,000 *g*. The supernatant containing gliadin was removed and the residue containing glutenin was resuspended in 500 μl of extraction buffer (50% propanol, 0.08 M Tris–HCl, pH 8.0) containing 2% dithiothreitol (DTT). The samples were incubated for 30 min at 65°C. After a 5 min centrifugation, 500 μl of extraction buffer containing 1.4% 4-vinylpyridine for alkylation was added to the supernatant. After incubation for 15 min at 65°C and centrifugation for 5 min, the supernatant was transferred to a new tube and stored at −80°C until use.

All extractions were performed at least two times.

### Analysis of Gliadin Using MALDI-TOF-MS

Two matrices were prepared according to the double layer method of instrument maker’s manual using sinapic acid (SA). Matrix I consisted of SA saturated in EtOH at a concentration of 10 mg/500 μl and matrix II consisted of SA saturated in 0.3% trifluoroacetic acid (TFA) in 50% acetonitrile (ACN) at a concentration of 200 mg/10 ml. Matrix I (1 μl) was spotted onto a MSP 96 target polished steel (Bruker Daltonics, Bremen, Germany) and allowed to air dry for approximately 5 min at room temperature. Each sample (1 μl) was diluted into 50 μl of matrix II and 1–1.5 μl of the sample/matrix II mixture was deposited onto the top of the matrix thin layer and then dried at room temperature. Gliadins were measured on a MALDI Microflex LT instrument equipped with a 60 Hz nitrogen laser (Bruker Daltonics, Bremen, Germany). Mass spectra were recorded in the linear positive mode and externally calibrated using a mixture of peptide/protein standards. To increase detection sensitivity, the following conditions were used: mass range 22,491–61,376 Da, sample rate 1.00 GS/s, laser shots 100, laser power 85%, laser frequency 60, and detector gain 33X. Each extracted sample was analyzed at least five times.

### Analysis of Gliadins Using RP-HPLC

Gliadin fractions were analyzed by RP-HPLC using a Waters Alliance e2695 equipped with an Agilent ZORBAX 300SB-C18 column (5 μm, 4.6 × 250 mm i.d., Agilent Technologies, United States). Water and ACN, both containing 0.1% TFA, were used as the mobile phase A and B. Dried gliadin pellets were mixed completely in 500 μl of 0.1% TFA in 20% ACN and filtered using a PVDF syringe filter (0.45 μm, Whatman, Maidstone, United Kingdom). Ten μl of each sample was injected and a linear gradient of  25–50% of solvent B was applied. The RP-HPLC analysis of gliadin was carried out with a flow rate of 1 ml/min at a column oven temperature of 65°C and monitored at a wavelength of 210 nm. Each extracted sample was analyzed at least five times.

## Results

### Sample Optimization for MALDI-TOF-MS

**FIGURE 1:**
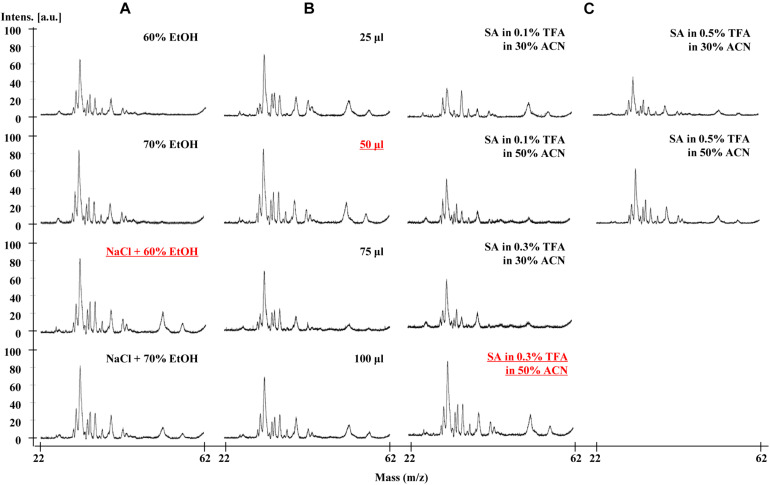
Effects of three major factors on MALDI-TOF-MS resolution of Chinese Spring gliadins. **(A)** gliadin extraction solvents, **(B)** dilution volume, and **(C)** matrix II components. The optimal conditions showing good resolution are underlined.

To optimize the resolution of the gliadin proteins in MALDI-TOF-MS analysis, we extensively tested three major factors. These included extraction solvents, dilution volumes, and composition of matrix II for protein ionization (Table 1 and Figure 1).

**TABLE 1:** Major factors used in this study to optimize mass spectra of MALDI-TOF-MS in gliadin fraction of Chinese Spring (CS).

Factor	Parameter
Extraction solvents	60% EtOH
	70% EtOH
	0.15 M NaCl + 60% EtOH
	0.15 M NaCl + 70% EtOH
Dilution volume	10 μl
	50 μl
	75 μl
	100 μl
Matrix II components	SA dissolved in 0.1% TFA in 30% ACN
	SA dissolved in 0.1% TFA in 50% ACN
	SA dissolved in 0.3% TFA in 30% ACN
	SA dissolved in 0.3% TFA in 50% ACN
	SA dissolved in 0.5% TFA in 30% ACN
	SA dissolved in 0.5% TFA in 50% ACN

### Extraction Solvents

To verify the effects of extraction solvents in mass spectra, we optimized the gliadin protein extraction from whole wheat grains according to the two previously reported methods (Dziuba et al., 2014; Han et al., 2015). The difference between the two methods is the use of 0.15 M NaCl to remove salt-soluble albumins and globulins before extracting gliadins with aqueous alcohol. Four extraction conditions were tested and compared: 60% EtOH, 70% EtOH, 0.15 M NaCl + 60% EtOH, and 0.15 M NaCl + 70% EtOH. Unexpectedly, two ω-5 gliadin peaks in the mass range of about 50–57 kDa were not detected in the gliadin fractions extracted with either 60 or 70% EtOH, but were present in those extracted with 0.15 M NaCl plus EtOH (Figure 1A). The best resolution was obtained from the gliadin fraction extracted with 60% EtOH after treatment with 0.15 M NaCl (Figure 1A).

### Dilution Volume

The effects of different dilution volumes (25, 50, 75, and 100 μl) were investigated to find the optimal volume of sample to dissolve in matrix II. As shown in Figure 1B, the mass spectra of the four dilution volumes were almost the same, and the 50 μl dilution volume was selected for MALDI-TOF-MS analysis and for reducing the amount of matrix II (Figure 1B).

### Matrix II and Optimization of Ratios of the Solvent

For matrix II, we added the ionization solvent SA, which is mainly used to ionize relatively high molecular weight proteins. TFA was added for sharpness of peaks and good resolution at concentrations of 0.1, 0.3, and 0.5%. We also optimized the amount of acetonitrile (ACN) (30% or 50%) in the MALDI-TOF-MS analysis of gliadin proteins. The mass spectra of gliadins with the solvent containing 50% ACN and 0.3% TFA with SA was found to be optimal (Figure 1C).

Based on the results described above, we concluded that the optimal method for sample preparation was first to extract gliadins with 60% EtOH containing 0.15 M NaCl. After mixing 1 μl of the gliadin mixture with 50 μl of a solvent of 50% ACN and 0.3% TFA with SA, 1 μl of the total mixture was loaded onto the target plate for MALDI-TOF-MS analysis.

### Analysis of Gliadin Proteins by MALDI-TOF-MS

**FIGURE 2:**
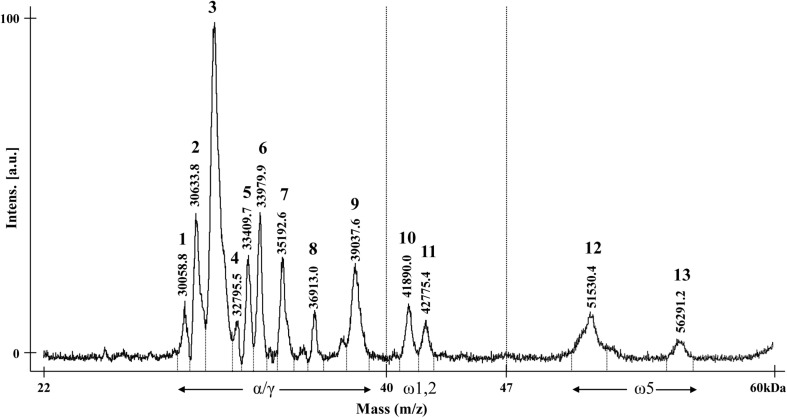
MALDI-TOF mass spectrum of gliadin fraction from Chinese Spring. The numbers for each gliadin peak and their observed molecular weights are indicated. Regions of α-, γ-, and ω-gliadin subgroups are shown with arrows.

The optimized method was used to analyze the gliadin profiles of CS wheat. The mass spectrum of gliadin extracts from CS showed 13 distinct peaks (Figure 2). The molecular ion signals of nine peaks at approximately 29–40 kDa, two peaks at approximately 40–44 kDa, and two peaks at approximately 50–57 kDa, likely correspond to α/γ-, ω-1, 2-, and ω-5 gliadins, respectively (Ferranti et al., 2007; Gil-Humanes et al., 2008; Sánchez-León et al., 2018).

**FIGURE 3:**
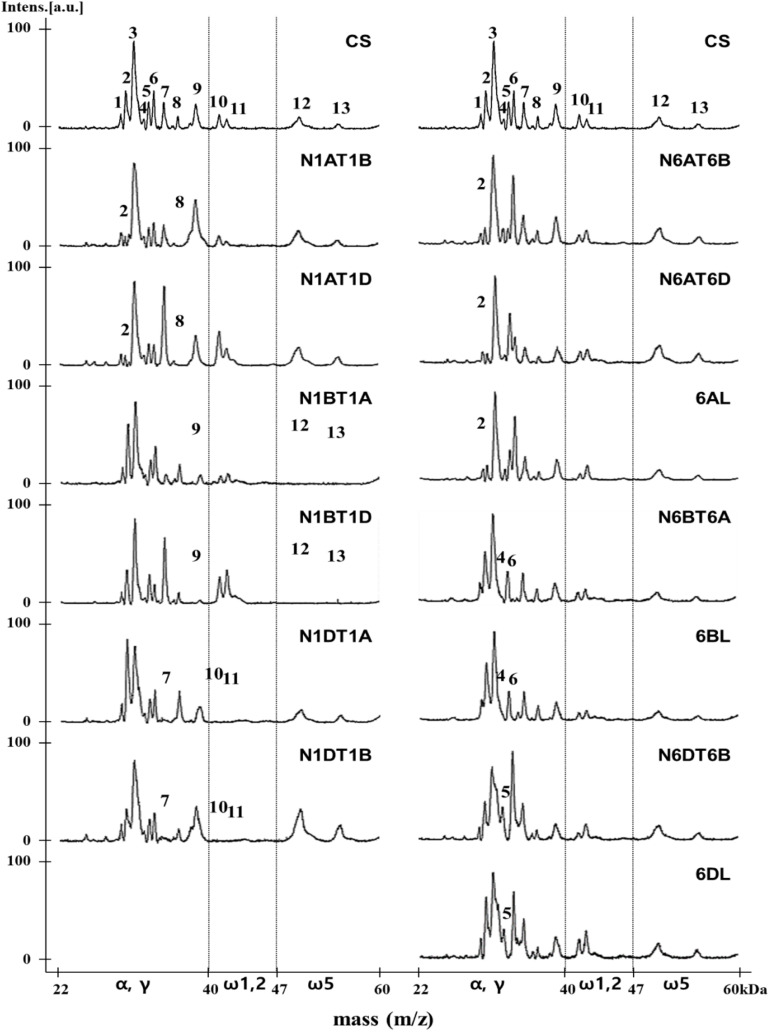
MALDI-TOF-MS profile analysis of gliadin fractions from Chinese Spring and its group 1 and 6 aneuploid lines to assign gliadin peaks to 1A, 1B, 1D, 6A, 6B, and 6D chromosomes. The numbers of distinctly reduced or missing peaks are marked on each mass spectrum of corresponding aneuploid lines of Chinese Spring.

Gliadins prepared from 10 nullisomic-tetrasomic (NT) lines for the group 1 or 6 chromosomes and three ditelosomic (DT) lines lacking the short arm of the group 6 chromosomes in the CS background were also analyzed in an attempt to assign proteins in peaks to specific chromosomes (Figure 3 and Table 2). Eight peaks (2, 7, 8, 9, 10, 11, 12, 13) were associated with the *Gli-A1*, *Gli-B1*, and *Gli-D1* loci. In the mass spectra of aneuploid lines N1AT1B and N1AT1D (which lack chromosome 1A), Peak 2 was drastically decreased while Peak 8 disappeared. Thus, these two peaks likely correspond to γ-gliadins on chromosome 1A. This assignment was supported by our findings that Peaks 2 and 8 increased in lines N1BT1A and N1DT1A (which lack chromosomes 1B and 1D, respectively, but have an extra copy of chromosome 1A). Peak 9 was significantly reduced in the α/γ-gliadin region and Peaks 12 and 13 disappeared completely in the ω-5 gliadin region in lines N1BT1A and N1BT1 (missing chromosome 1B). Because these peaks also increased in lines N1AT1B and N1DT1B (containing an extra chromosome 1B), they were assigned to chromosome 1B. Peak 7 in the α/γ-gliadin region and Peaks 10 and 11 in the ω-1,2 gliadin region were assigned to chromosome 1D by comparing the mass spectra of lines N1DT1A and N1DT1B (missing chromosome 1D) and N1AT1D and N1BT1D (containing an extra copy of chromosome 1D).

**TABLE 2:** Characteristics of 13 gliadin peaks resolved by MALDI-TOF-MS in Chinese Spring and its aneuploid lines, comparison with proteins deduced from full-length gliadin genes by Huo et al(2018a; 2018b), and with results of quantitative 2-DE analysis reported by Altenbach et al. (2020).

MALDI-TOF-MS				Huo et al., 2018a,b		Altenbach et al., 2020	
Peak	Range^*a*^	Chromosome^*b*^	Gliadin^*c*^	Gene^*d*^	Predicted MW^*e*^	2-DE Spot#	Spot volume^*f*^
1	29800–30300	Unknown		α-A10	29,996	117,118	0.8
				α-D12	30,175		
2	30300–31200	1A	γ	α-A4	30,506	119,120	1.8
		6A	α	α-A6	30,621	121,122,123	0.5
				γ-A3^*g*^	30,655	123, 124,125,126	2.9
				γ-A4^*g*^	30,655	123,124,125,126	2.9
				α-D1	30,699		
				α-D9	30,810		
				γ-B6	30,982	121,122,123,139	0.8
				α-A8	31,050		
3	31200–32500	Unknown		α-B15	31,284		
				α-B14	31,413	109,112	0.7
				α-D8^*h*^	31,435	84,85,107,111,113,114	2.1
				α-A1	31,440	111,113,114	1.4
				α-B11	31,535		
				α-D4	31,542	108,109	1.4
				γ-D3	31,619	109	0.3
				α-D6^*h*^	31,706	102	0.4
				α-B18	31,829		
				γ-B2	31,834	102	0.4
				γ-D4	31,865		
				α-B17	32,039	82	0.2
				α-B16	32,054	113	0.1
				α-A9	32,181		
				γ-B1^*h*^	32,431	99	0.4
4	32500–33050	6B	α	γ-D1^*h*^	32,606	99,100,102,107	1.1
5	33050–33600	6D	α	α-D5	33,412	103,106,110,112	1.7
				α-A5	33,479		
6	33600–34300	6B	α	α-B25	33,818		
				α-B7	33,968	85,104	0.5
				α-B9	33,977	105	0.9
NP	34300–34900			α-A2	34,471	81,82,83,84	0.8
				δ-B1	34,558		
				α-B8	34,781	86,91	0.9
7	34900–35900	1D	γ	γ-D2	35,188	86,101	1.7
				δ-D1	35,450	80.81.82	0.9
NP	35900–36600			α-B3	36,206	62	0.3
8	36600–37300	1A	γ	γ-A1	36,892	61.77.78	1.9
9	38600–39900	1B	γ	ω-A4^*i*^	39,651	37,75,79	1.1
				γ-B4	39,892	74,75,76,78	1.9
10	41550–42400	1D	ω-1,2	c^*h,i*^	41,831	34,35	0.5
11	42400–43200	1D	ω-1,2	ω-D2^*g,i*^	42,744	31,32	3.5
				ω-D3^*g,i*^	42,744	31,32	3.5
NP	43200–50500			ω-D4^*j*^	44,416	43	0.3
				ω-B3	47,650		
12	50500–52000	1B	ω-5	ω-B6	51,532	25,27,28,29	6.3
13	55600–56800	1B	ω-5				

*^*a*^Mass range from the beginning to the end of each peak as shown in Figure 2. Mass ranges not associated with specific peaks are designated as NP in column 1. ^*b*^Assigned chromosome determined by comparing MALDI-TOF-MS profiles of Chinese Spring and its aneuploid lines. ^*c*^Assigned gliadin protein determined by comparing MALDI-TOF-MS profiles of Chinese Spring and its aneuploid lines, and by mass range. ^*d*^Gliadin genes that encode full-length proteins within the mass range of each peak. ^*e*^Mature protein following removal of signal peptide. ^*f*^% total normalized volume determined by quantitative 2DE analysis. ^*g*^Genes encode identical proteins; the same spot numbers and % total normalized volume are reported for both genes. ^*h*^Genes encode proteins containing an odd number of cysteine residues that are likely to partition in the glutenin fraction. ^*i*^The second predicted MW as a result of processing with an asparingyl protease is not indicated. ^*j*^Considered pseudogenes in Huo et al., 2018a but they contained stop codon near 3′ end resulting in truncated proteins reported by Altenbach et al., 2018, 2020.*

The comparison of MALDI-TOF mass spectra of the group 6 aneuploid lines missing genes encoding α-gliadins with that of CS allowed the assignment of four peaks (2, 4, 5, 6) to the *Gli-2* loci (Figure 3 and Table 2). In the mass spectra of lines N6AT6B, N6AT6D, and 6AL (which lack chromosome 6A or the short arm of chromosome 6A (6AS), Peak 2 was very small, whereas it was increased in line N6BT6A (containing an extra copy of chromosome 6A), indicating that Peak 2 corresponds to α-gliadins on chromosome 6A. Considering the results in the previous paragraph, gliadins in Peak 2 were assigned to two chromosomes, 1A and 6A. The profiles of lines N6BT6A and 6BL (which are missing chromosome 6B or 6BS), enabled us to assign Peaks 4 and 6 to chromosome 6B. This assignment was also supported by an increase of the two peaks in line N6DT6B (containing an extra chromosome 6B). Peak 5 was assigned to chromosome 6D by analyzing the mass spectra of lines N6DT6B and 6DL (missing chromosomes 6D or 6DS, respectively), and N6AT6D (containing an extra copy of chromosome 6D). Of the 13 peaks in MALDI-TOF mass spectra for CS, 10 peaks were assigned to either chromosomes 1 or 6, one peak (2) was assigned to both chromosomes 1 and 6, and two peaks (1, 3) could not be assigned.

The availability of the sequences for the complete set of full-length gliadin genes from CS (Huo et al., 2018a,b) made it possible to compare the observed molecular weights (MWs) of peaks in the CS gliadin fraction determined by MALDI-TOF-MS to the predicted MWs of the mature proteins. Table 2 shows the mass ranges corresponding to the beginning and end of peaks observed in MALDI-TOF-MS (Figure 2). Gliadin proteins with predicted MWs in the mass range of each peak are also shown along with their accumulation levels in wheat flour measured by quantitative 2-DE analysis combined with MS/MS (Altenbach et al., 2020).

Peak 1 was a minor peak in the MALDI-TOF spectrum that could not be assigned to any chromosome. Two CS gliadins fell in the mass range of this peak, α-A10 and α-D12. However, only α-A10 was also observed by 2-DE. Peak 2 was assigned to chromosomes 1A and 6A. Five α-gliadins (α-A4, α-A6, α-A8, α-D1, and α-D9) and three γ-gliadins (γ-A3, γ-A4, and γ-B6) fell in the mass range of this peak. However, α-gliadins encoded by chromosome 6D were not observed in 2-DE and the gliadins encoded on chromosome 1A and 6A encompassed the bulk of the 2-DE spot volume, consistent with the chromosomal assignment. Fifteen CS gliadins fell within the mass range of peak 3, although three (α-D6, α-D8, and γ-B1) contained an odd number of cysteine residues and likely partition into the glutenin fraction rather than the gliadin fraction. Two, 6 and 2 of the remaining α-gliadins were from the 6A, 6B, and 6D chromosomes, respectively, while 1 and 2 of the remaining γ-gliadins were from the 1B and 1D chromosomes, respectively. Not surprisingly, it was not possible to assign this peak to a particular chromosome using the aneuploid lines. Peak 4 was a very minor peak that was assigned to chromosome 6B. However, the only protein that fell into its mass range was γ-D1 containing an odd number of cysteine residues. Peak 5 was assigned to chromosome 6D and has two gliadin proteins in its mass range, α-A5 and α-D5. However, α-A5 was not observed in the 2-DE analysis. Interestingly, α-D5 is the only α-gliadin in CS that contains the 33-mer toxic peptide, a major trigger for celiac disease (Shan et al., 2002). Gliadins that fell within the mass ranges of peaks 6, 7, 8 were consistent with their chromosomal assignments. Peak 9 was assigned to chromosome 1B, however, both γ-B4 and ω-A4 fell into its mass range. γ-B4 accounted for a greater proportion of the spot volume in quantitative 2-DE. ω-gliadins that fell within the mass ranges of peaks 10, 11 and 12 were consistent with their chromosomal assignments although it should be noted that ω-D1 contains a single cysteine residue. Peak 13 was assigned to chromosome 1B, but there was no gliadin protein corresponding to its mass range. However, it is interesting that one of the four protein spots identified by MS/MS as ω-B6 had a notably slower mobility in 2-DE than the other spots (Altenbach et al., 2020).

### Analysis of Gliadin Proteins by RP-HPLC

**FIGURE 4:**
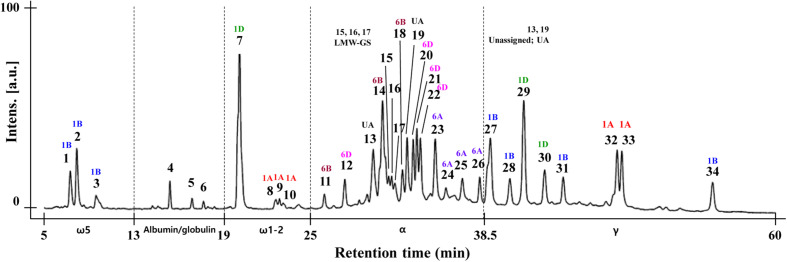
RP-HPLC analysis of gliadin fraction from Chinese Spring. The eluted peak number and the result of chromosomal assignment of individual peaks by comparing the RP-HPLC pattern with group 1 and 6 aneuploid lines are indicated. The position of albumin/globulin proteins (Peaks 4, 5, 6), contaminated LMW-GS (Peaks 15, 16, 17) and unassigned peaks (UA, Peaks 13, 19) are shown as explained in section “Analysis of Gliadin Proteins Using RP-HPLC” of the Results.

Gliadin fractions from CS also were extracted and analyzed by RP-HPLC with resolution optimized by adjusting elution time, temperature, flow rate, and solvent conditions as described in the Materials and Methods. As shown in Figure 4, **34** peaks from CS were eluted according to their hydrophobicity. To ensure that the gliadins were not contaminated with other proteins, the glutenin fraction of CS was extracted using 50% propanol, and RP-HPLC was conducted under the same analytical conditions. The chromatograms of the gliadin and glutenin fractions were compared. Peaks 15, 16, and 17 were identified as likely LMW-GS (Supplementary Figure 1). Similarly, to identify possible contamination of the extract with salt-soluble albumins/globulins, gliadin proteins were extracted with 70% EtOH and 70% EtOH containing 0.15 M NaCl. Comparison of the chromatograms from the two extraction methods showed that Peaks 4, 5, and 6 were likely albumins/globulins (Supplementary Figure 1). Thus, ω-5 gliadins eluted in three peaks (1–3) between 5 and 13 min, and ω-1,2 gliadins eluted in four peaks (7–10) between 19 and 25 min. α-gliadins were between 25 and 38.5 min and separated into 13 peaks (11–14, 18–27). γ-gliadins eluted in seven peaks (28–34) between 39 and 60 min (Figure 4).

**FIGURE 5:**
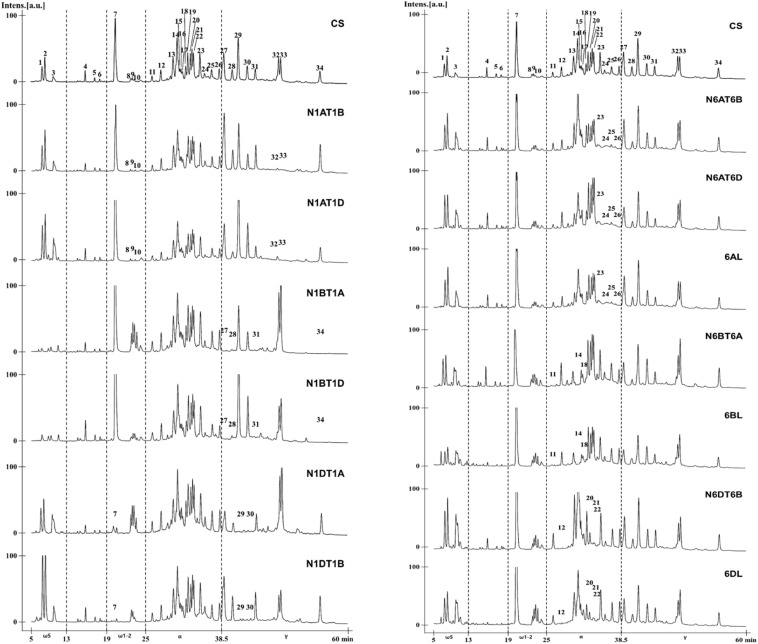
RP-HPLC pattern analysis of gliadin fractions from Chinese Spring and its group 1 and 6 aneuploid lines to assign gliadin peaks to 1A, 1B, 1D, 6A, 6B, and 6D chromosomes. The numbers of distinctly reduced or missing peaks are marked on the chromatogram for the corresponding aneuploid lines of CS.

By comparing the chromatogram patterns of RP-HPLC of CS and its aneuploid lines for group 1 and 6 chromosomes, the positions of gliadin peaks encoded by each chromosome were determined (Figure 5). To identify gliadin peaks encoded by chromosome 1A, we compared the chromatograms of CS with those of lines N1AT1B and N1AT1D. Peaks 8–10 of ω-1,2 gliadin were reduced, leaving only traces, and peaks 32 and 33 of γ-gliadin disappeared. The conclusion that peaks 8, 9, and 10 of ω-1,2 gliadin are encoded by chromosome 1A was also supported by an increase of these three peaks in the 1A tetrasomic lines, N1BT1A and N1DT1A. For detection of gliadin proteins encoded by chromosome 1B, we analyzed the RP-HPLC profiles of CS and the chromosome 1B aneuploid lines, N1BT1A and N1BT1D, and observed that peaks 1, 2, and 3 of ω-5 gliadin were reduced, leaving only traces, and peaks 27, 28, 31, and 34 of γ-gliadin were absent. The traces of peaks 1, 2, and 3 in lines N1BT1A and N1BT1D were assumed to be ω-5 gliadins encoded by ω-D4 on chromosome 1D rather than chromosome 1B, as its expression level is very low (Altenbach et al., 2018, 2020; Huo et al., 2018a). Lines N1DT1A and N1DT1B were used to assign gliadin peaks encoded by chromosome 1D. In these lines, peak 7 of ω-1,2 gliadin and peaks 29 and 30 of γ-gliadin were almost absent. Therefore, among chromosome 1A- and 1D-encoded ω-1,2 gliadins in CS, chromosome 1D encodes the most abundant form.

The NT and DT lines deficient in chromosomes 6A/6AS, 6B/6BS, and 6D/6DS were used to assign gliadin peaks to chromosome 6 (Figure 5). Comparison of RP-HPLC profiles of N6AT6B, N6AT6D, and 6AL (null 6A/6AS) lines with that of CS revealed that peaks 23, 24, 25, and 26 of α-gliadin were associated with chromosome 6A. In lines N6BT6A and 6BL (which lack chromosome 6B/6BS), peaks 11, 14, and 18 were absent in the α-gliadin region. In the profiles of lines N6DT6B and 6DL (which lack chromosomes 6D/6DS), peaks 12, 20, 21, and 22 were absent. When combining the comparative analysis of all RP-HPLC profiles, only two peaks (13 and 19) out of 35 peaks could not be assigned to group 1 or 6 chromosomes.

As shown in Figure 4, the area of ω- 5-, ω-1, 2-, and γ-gliadin peaks were well separated, but peaks 13–22, mainly assigned to chromosomes 6B and 6D α-gliadin, were difficult to separate. This difficulty can be explained by the grand average of the hydropathicity index (GRAVY) of the 28 full-length gliadin genes of CS with a 2-DE spot volume of 0.4 or higher (Supplementary Table 1). The GRAVY is used to represent the hydrophobicity value of a peptide and calculates the sum of the hydropathy values for all the amino acids divided by the sequence length. From these data, it can be seen that the GRAVY was very similar in each of the α-gliadins derived from chromosomes 6A, 6B, and 6D compared to other gliadin subgroups.

## Discussion

MALDI-TOF-MS and RP-HPLC procedures were optimized and assessed for use as automatable, high-throughput methods for screening wheat lines with altered gliadin compositions. MALDI-TOF-MS separates proteins on the basis of molecular mass and is attractive because the complete analysis of gliadin proteins in a sample can be completed in about 1 min. RP-HPLC, on the other hand, separates proteins on the basis of hydrophobicity and has been used to analyze gliadin fractions in wheat flours for many years (Brown and Flavell, 1981; Bietz, 1983; Bietz and Burnouf, 1985; Liu et al., 2005; Han et al., 2015; Sánchez-León et al., 2018). However, analysis time per sample is considerably greater than MALDI-TOF-MS, about 70 min per sample.

Both ω-5 and ω-1,2 gliadins were easily resolved by MALDI-TOF-MS. Since ω-5 gliadins contain numerous epitopes that trigger WDEIA and ω-1,2 gliadins contain immunodominant epitopes for CD, both are likely targets for efforts to reduce immunogenic potential of the flour. In comparison, it was difficult to separate individual α- and γ-gliadins because many of the proteins had very similar molecular masses. This represents a fundamental limitation of the MALDI-TOF-MS method. In fact, in one case a single peak in the spectrum likely contained as many as 12 α- and γ-gliadins. Because these gliadins were encoded on chromosomes 6A, 6B, 6D, 1B and 1D, it was also impossible to assign this peak to a single chromosome. This suggests that it would be difficult to detect deletions of multiple α- and γ-gliadin genes on a single chromosome by MALDI-TOF-MS. Nonetheless, three of the nine gamma gliadins that were identified by 2-DE combined with MS/MS in Altenbach et al. (2020) were distinguished by MALDI-TOF-MS. All were relatively abundant proteins encompassing from 1.7 to 1.9% of the total flour protein. Each protein also contained 10 CD epitopes, the most among the CS γ-gliadins. As a result, these γ-gliadins may be the focus of targeted efforts to reduce the immunogenicity of the flour. Only three of 16 α-gliadins identified in 2-DE could be distinguished by MALDI-TOF-MS. It is notable that these included α-D5 in peak 5, and α-B7 and α-B9 in peak 6. Because α-D5 contains the 33-mer toxic peptide, this protein is also a likely candidate for targeted efforts to reduce the immunogenicity of the flour. In comparison, α-B7 and α-B9 do not contain CD epitopes and the presence of this peak in a sample could provide evidence that efforts to selectively eliminate only those proteins containing abundant CD epitopes were effective. Thus, MALDI-TOF-MS could be useful as a rapid screening method, depending on the overall goals of the experiment. Thus far, MALDI-TOF-MS has been used to screen wheat lines in two studies, one that used RNAi (Gil-Humanes et al., 2008) and one that used gene editing to reduce immunogenic potential (Sánchez-León et al., 2018).

Like MALDI-TOF-MS, RP-HPLC effectively separated both ω-5 and ω-1,2 gliadins. Additionally, proteins in the α- and γ-gliadin families were separated by RP-HPLC and a greater number of proteins within each family were resolved. Of the 13 peaks assigned to α-gliadins, four were assigned to chromosome 6A, three to 6B, four to 6D and two were unassigned. Of the 8 peaks assigned to γ-gliadins, two were from chromosome 1A, four from 1B, and two from 1D. In comparison, 16 and 9 protein spots were identified as α- and γ-gliadins, respectively, in quantitative 2-DE experiments of CS (Altenbach et al., 2020). Thus, the RP-HPLC profile of the gliadin fraction generated in this study revealed most of the major gliadin proteins expressed in CS wheat flour. Although most peaks could be assigned to individual chromosomes using the CS aneuploid lines, it was not possible to discern which proteins were found in each peak, a potential drawback of RP-HPLC. Further analysis of peaks by MALDI-TOF-MS or MS/MS is therefore required to identify individual proteins. In the final analysis, it is probably easier to detect overall changes in α- and γ-gliadins by RP-HPLC than MALDI-TOF-MS, despite the longer analysis time. If chromosomal assignments can be made, it should also be possible to detect deletions of multiple gliadin genes on a single chromosome. However, it is important to keep in mind that aneuploid lines are not available for many cultivars.

Undoubtedly, experiments aimed at reducing the immunogenic potential of flour will be conducted in commercial cultivars grown in various parts of the world rather than in the reference cultivar CS. Because of tremendous allelic variation among cultivars, the complement of gliadins in each cultivar will need to be evaluated. Fortunately, the availability of a reference sequence from CS makes it possible to use gene capture methods to obtain the sequences of gliadins from many different cultivars. Analyses of these sequences will reveal the number of epitopes for CD and food allergies in each protein and highlight the best approaches to reduce the immunogenic potential of the flour. Only then will it be possible to determine whether MALDI-TOF-MS or RP-HPLC is most appropriate for screening altered lines. In any event, it is important to keep in mind that such analyses are only the first screening step and that more robust, time-consuming, and technically challenging methods such as 2-DE combined with MS/MS must ultimately be used to thoroughly characterize the selected lines.

## Data Availability Statement

The raw data supporting the conclusions of this article will be made available by the authors, without undue reservation.

## Author Contributions

J-YL, Y-RJ, and KC designed and conducted the experiments, and wrote the manuscript. YG, SA, T-WG, B-GK, and S-HL contributed to the interpretation of data. SK, J-RS, and S-BL conducted the experiments. All authors contributed to the article and approved the submitted version.

## Conflict of Interest

The authors declare that the research was conducted in the absence of any commercial or financial relationships that could be construed as a potential conflict of interest.

## Supplementary Material

Supplementary Figure 1 RP-HPLC pattern analysis of two gliadin extraction methods (70% EtOH and 0.15 M NaCl + 70% EtOH) and glutenin fraction to confirm salt soluble albumin/globulin (Peaks 4, 5,6) and contaminated LMW-GS (Peaks 15, 16, 17).

Supplementary Table 1 Grand average of hydropathicity index (GRAVY) for 28 full-length gliadin proteins of Chinese Spring with 2-DE spot volume of 0.4 or higher.
