# Supplementary figures and images for: Comparison of MALDI-TOF-MS and RP-HPLC as Rapid Screening Methods for Wheat Lines With Altered Gliadin Compositions
Source: Front Plant Sci. 2020 Dec 4;11:600489. doi: 10.3389/fpls.2020.600489 (PMC7746642; doi:10.3389/fpls.2020.600489)

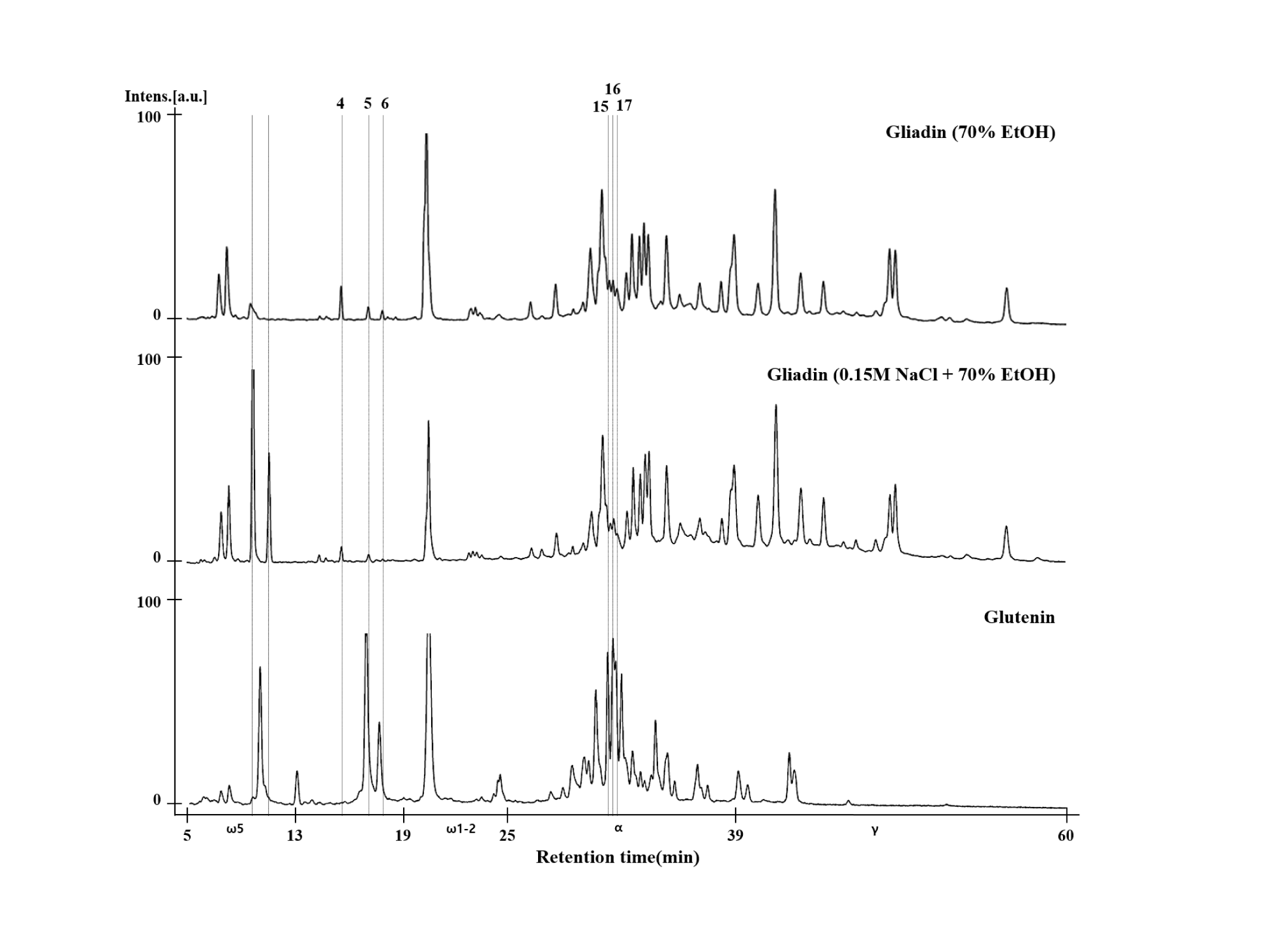

Supplement: Supplementary Figure 1 — RP-HPLC pattern analysis of two gliadin extraction methods (70% EtOH and 0.15 M NaCl + 70% EtOH) and glutenin fraction to confirm salt soluble albumin/globulin (Peaks 4, 5,6) and contaminated LMW-GS (Peaks 15, 16, 17). [file Image_1.TIF]
